# Inducible *Slc4a11* Knockout Triggers Corneal Edema Through Perturbation of Corneal Endothelial Pump

**DOI:** 10.1167/iovs.62.7.28

**Published:** 2021-06-30

**Authors:** Diego G. Ogando, Rajalekshmy Shyam, Edward T. Kim, Yen-Chiao Wang, Chia-Yang Liu, Joseph A. Bonanno

**Affiliations:** 2 Vision Science Program, School of Optometry, Indiana University, Bloomington, Indiana, United States

**Keywords:** barrier function, corneal endothelial dystrophy, lactate transporters, oxidative stress

## Abstract

**Purpose:**

The conventional *Slc4a11* knockout (KO) shows significant corneal edema at eye opening, a fact that complicates the study of the initial events leading to edema. An inducible KO would provide opportunities to examine early events following loss of Slc4a11 activity.

**Methods:**

*Slc4a11 Flox* (SF) mice were crossed with mice expressing the estrogen receptor Cre Recombinase fusion protein and fed tamoxifen (Tm) for two weeks. Corneal thickness (CT) was measured by OCT. At eight weeks endpoint, oxidative damage, tight junction integrity, stromal lactate concentration, endothelial permeability, differentially expressed transporters, and junction proteins were determined. Separately, a keratocyte only inducible *Slc4a11* KO was also examined.

**Results:**

At four weeks post-Tm induction *Slc4a11* transcript levels were 2% of control. Corneal thickness increased gradually and was 50% greater than Wild Type (WT) after eight weeks with significantly altered endothelial morphology, increased nitrotyrosine staining, significantly higher stromal lactate, decreased expression of lactate transporters and Na-K ATPase activity, higher ATP, altered expression of tight and adherens junctions, and increased fluorescein permeability. No significant differences in CT were found between WT and keratocyte only *Slc4a11* KO.

**Conclusions:**

The *Slc4a11* inducible KO shows development of a similar phenotype as the conventional KO, thereby validating the model and providing a tool for further use in examining the sequence of cellular events by use of noninvasive in vivo physiological probes.

Slc4a11 mutations are associated with congenital hereditary endothelial dystrophy (CHED).^1^ CHED is an autosomal recessive disorder that presents corneal edema and loss of corneal endothelial cells within the first decade of life.^2^ In this pathology, corneal opacification is observed due to stromal and epithelial edema and thickening of the Descemet's membrane.^3^ In a study performed in Spain in 1998, the incidence of CHED was reported to be 3.11/100,000 newborns.^4^ CHED is typically bilateral, the severity often asymmetric and accompanied by nystagmus.^5^ The current treatments for this disease are Descemet stripping automated endothelial keratoplasty (DSAEK) and Descemet membrane endothelial keratoplasty (DMEK). *Slc4a11* KO mice recapitulate CHED. In these mice, significant corneal edema is present from eye opening in the absence of significant loss in endothelial cell density.^6^^,^^7^ Corneal thickness and stromal lactate are doubled in the KO at 12 weeks and both are tripled at 40 weeks of age^7^ consistent with the linkage between lactate efflux and endothelial pump activity.^8^ As corneal edema progresses, apoptotic cell death accelerates significantly diminishing corneal endothelial (CE) density.^6^^,^^7^ Since *Slc4a11* is null during embryonic development and corneal edema is present at eye opening, unraveling the initial events that lead to corneal edema would benefit by an inducible KO.

Recently, we reported that Slc4a11 is an NH_3_-activated mitochondrial uncoupler that facilitates glutamine catabolism and suppresses mitochondrial superoxide production.^9^ Cells lacking Slc4a11 have a hyperpolarized mitochondrial membrane potential that results in increased O_2_^−^ production and oxidative damage. In vitro, these cells have decreased glutaminolysis capacity^10^ and show increased mitochondrial oxidative stress and apoptosis in the presence of glutamine.^9^ A comparative transcriptome analysis performed in CHED primary CE cells and a mouse corneal endothelial cell line derived from *Slc4a11* KO mice indicated a generalized inhibition of metabolic and transport gene expression in KO cells.^11^ Oxidative stress appears to be a root cause of these changes since quenching mitochondrial ROS, mitochondrial uncoupling of KO cells, or circumventing glutaminolysis, all of which lower ROS production, rescue KO cells, and reverse corneal edema progression.^9^

In this study we established an inducible *Slc4a11* KO mouse model (whole body KO) that presents progressive changes in corneal thickness. In this mouse we found endothelial oxidative stress and significant alterations in endothelial pump function, similar to that seen in the conventional KO. The inducible *Slc4a11* KO will provide opportunities to examine the very earliest events of cell and tissue dysfunction that lead to gene expression changes, the development of corneal edema, and eventual apoptosis.

## Materials and Methods

### Mice and Treatments

**Figure 1.**
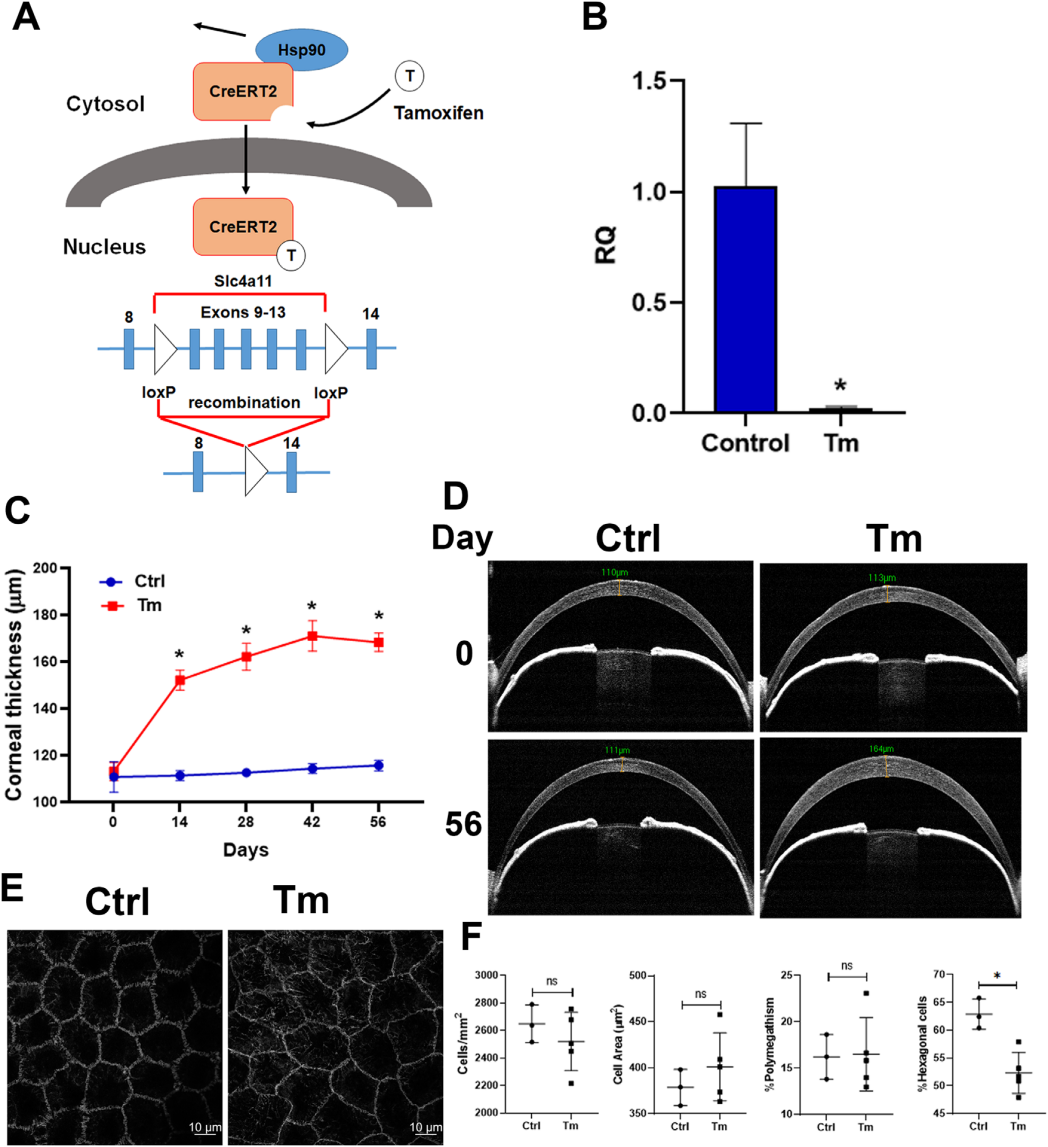
Inducible *Slc4a11* KO induces gradual corneal edema. **A**. Schematic of the inducible *Slc4a11* KO strategy. **B**. Relative quantification (RQ) of *Slc4a11* in corneal tissue at four weeks of Tm (tamoxifen) treatment by QPCR; mean ± SEM, *n* = 4, *P < 0.05. **C**. Corneal thickness versus time after Tm treatment; mean ± SEM, *n* = 4, *P < 0.05. **D**. Representative OCT pictures of Ctrl (control) and Tm corneas. **E**. Cell shape delineated by ZO-1 staining after eight weeks of Tm treatment. **F**. Cell morphology analysis eight weeks after Tm treatment. *n* = 3 for Ctrl, *n* = 5 for Tm; mean ± SEM, n = 5, *P < 0.05.

*Slc4a11* Flox/Flox that contains loxP enclosing exons 9–13 were obtained from Ozgene (Fig. 1A). B6.129-Gt(ROSA)26Sortm1(cre/ERT2)Tyj/J mouse line (Stock No: 008463) expressing the estrogen receptor-Cre Recombinase fusion protein: Cre-ERT2 were obtained from Jackson Laboratories. Cre-ERT2 is under a strong promoter and expected to be expressed by all tissues. *Slc4a11^Flox/^^Flox^*//*Rosa^Cre-ERT2^*^/^*^Cre-ERT2^* mice at eight weeks of age were fed with tamoxifen (Tm) enriched chow (0.4g/kg) for two weeks, followed by normal chow. These mice are expected to be whole body *Slc4a11* KO. Controls of the same genotype were fed normal chow throughout. WT mice were fed Tm chow for two weeks to check for nonspecific effects of tamoxifen on corneal thickness.

Genotpying for *Slc4a11* flox and WT alleles was performed using primers: Forward: TCTGGACTTCAACGCCTTCT and Reverse: GCACAAACGTGATGGAAATG. WT allele is 353 bp and flox allele is 438 bp. PCR consist on 94°C for two min followed by 35 cycles of 94°C for 30 seconds, 55°C for 30 seconds and 68°C for one minute.

Genotyping for Cre-ERT2 and WT alleles was performed using primers: Forward: AAAGTCGCTCTGAGTTGTTAT and Reverse: GGAGCGGGAGAAATGGATATG for WT allele of 603 bp and Forward: AAAGTCGCTCTGAGTTGTTAT and Reverse: CCTGATCCTGGCAATTTCG for Cre-ERT2 allele of 825 bp. PCR consists of 94°C for two minutes, followed by a touchdown phase of 10 cycles of 94°C for 20 seconds, 65°C for 15 seconds (–0.5°C per cycle decrease) and 68°C for 10 seconds. This was followed by 28 cycles of 94°C for 15 seconds, 60°C for 15 seconds, and 72°C for 10 seconds. Followed by 72°C for two minutes.

Mice engineered to be inducible *Slc4a11* KO strictly in keratocytes were produced: *Slc4a11* Flox mice were crossed with mice expressing doxycycline receptor rtTA under Keratocan promoter (*Kera-rtTA*) and with mice expressing Cre recombinase under the doxycycline response element (*tetO-Cre*). The use of this keratocyte specific driver was described previously.^12^ *Slc4a11^Flox/^^Flox^*//*Kera-rtTA*//*tetO-Cre* mice and control littermates *Slc4a11^WT^*//*Kera-rtTA*//*tetO-Cre* were fed with doxycycline since embryonic day 0 until the day of the experiment at three months of age.

The knock-in KeraRT mice were identified by PCR using the following PCR primers: Kera-F1 (primer #5), 5-TGGTGGCTTGCTTCAAGCTTCTTC-3 and reverse primer Kera-R1 (primer #6), 5 -TATCCAACTCACAACGTGGCACTG-3, for knock-in allele of 461 bp; and Kera-F1 and Kera-R2 (primer #7), 5-GGAGTCTGCACTACCAGTACTCAT-3 for WT allele of 389 bp. The PCR protocol was as follows: 98°C for 30 seconds, 35 cycles of 98°C for 30 seconds, 65°C for 30 seconds, and 70°C for 15 seconds. This was followed by 70°C for five minutes.

All mice were housed and maintained in pathogen-free conditions and used in the experiments in accordance with institutional guidelines and the current regulations of the National Institutes of Health, the United States Department of Agriculture and Association for Research in Vision and Ophthalmology (ARVO) Statement for the Use of Animals in Ophthalmic and Vision Research.

### Optical Coherence Tomography

Central corneal thickness was measured using optical coherence tomography (iVue100 Optovue, Inc., Fremont, CA, USA) with anterior segment lens attachment, before treatment and biweekly after Tm feeding until endpoint at eight weeks posttreatment. Mice were anesthetized by intraperitoneal injection of solution containing ketamine 80 mg/kg and xylazine 5 mg/kg. Ocular surface was kept hydrated with Lacripure saline solution (Menicon America Inc.). Cornea pachymetry is performed by acquiring eight radial line scans containing 1024 A-scans each. The horizontal line scan is obtained eight times and then averaged. This averaged scan is used for corneal thickness measurement. The iVue software is designed for detecting the human cornea dimensions and cannot assign the boundaries of the mouse cornea. Corneal thickness was obtained at the corneal apex using a distance tool included in the software. The person performing the measurements was blinded about mouse treatment.

### Immunofluorescence

Corneal buttons were dissected and placed in 96 well plates. All steps were performed in PBSCM that is PBS, pH: 7.4 containing 1 mM CaCl_2_ and 1 mM MgCl_2_. Tissue was fixed for 10 min at room temperature in 4% paraformaldehyde in PBSCM. Then, washed two times for five minutes each in PBSCM. Cells were permeabilized in 0.5% (v/v) Triton X-100 in PBSCM. After two washes in PBSCM, tissues were blocked for one hour in 2% BSA in PBSCM. Primary antibodies: mouse anti-ZO-1 (1:100) (Thermo Fisher Scientific #33-9100), rabbit anti-Nitrotryrosine (1:200) (Thermo Fisher Scientific #A21285) were added in 2% BSA in PBSCM and incubated overnight at 4°C. Tissues were washed three times in PBSCM, incubated with secondary antibodies for one hour at room temperature and washed three times in PBSCM. F-actin was stained by adding Phalloidin-A488 and incubated for 20 minutes at room temperature. After three washes, corneal buttons were flattened by performing relaxing cuts and mounted on coverslips with one drop of Vectashield (#H1500) containing DAPI.

### Nitrotyrosine Intensity Quantification

Corneas were simultaneously stained for nitrotyrosine and ZO-1 as indicated above. Five images were acquired from each cornea using a Zeiss LSM 800 confocal microscope with 40× oil objective with identical exposure time, laser intensity, gain, and offset for all images. Using ImageJ, cell borders were drawn (including partial cells along left and bottom sides and excluding partial cells along the right and top sides of the square) and nitrotyrosine staining mean fluorescence intensity of each cell was obtained. Average mean intensity values of OD and OS corneas of each animal were averaged. Four mice per treatment were used.

### Cell Morphology Analysis

ZO-1 fluorescent staining was performed and five images acquired from each cornea using a 40× objective. Cell morphology analysis was performed with ImageJ as previously described by Behndig et al.^13^ Briefly, cell borders were drawn and cells were counted, including partial cells along left and bottom sides and excluding partial cells along the right and top sides of the square. Cell density was normalized to the area of each image in square millimeters. Cell areas were calculated by the software, and number of cell sides (number of neighbor cells) and percentage of hexagonal cells were calculated manually. Percent polymegathism was calculated as: standard deviation of cell area x100/mean cell area. Average values of OD and OS corneas of each animal were averaged. Three mice were used for control group and five mice for tamoxifen group.

### Total RNA Isolation from CEDM for RNA Sequencing of Conventional KO

RNA was isolated using AllPrep DNA/RNA Mini Kit. Kit (Qiagen #80204). Six CEDM peelings from three animals were pooled and disrupted in 300 µl of RLT Buffer plus β-Mercaptoetnanol using a disposable pestle. Samples were homogenized by applying to a QIAshredder spin column (Qiagen #79654). The flow through was applied to a AllPrep DNA column and centrifuged at 10,000 × *g* for 40 seconds. The column containing DNA was discarded. The flow through containing RNA was mixed with ethanol 100% (40% vol/vol) and applied to a RNAeasy Micro column. The column was centrifuged at 10,000 × *g* for 15 seconds. The flow through containing small RNA (<200 nucleotides) was discarded. The column was consecutively washed with RW1 buffer, RPE buffer, and 80% ethanol. RNA was eluted with 14 µl of nuclease-free water. RNA concentration and integrity were measured with Agilent 2200 TapeStation system. Only RNA of RNA Integrity Number > 6 were used for further processing. Five WT and five KO pools were processed.

### RNA Sequencing of Total RNA

The RNAseq libraries were generated using the TruSeq Stranded mRNA kit protocol. In brief, first the integrity of the RNA was checked with Agilent TapeStation; samples with RIN > 7 were used for library preparation. The mRNA was purified from 100 ng of total RNA for each sample, after cDNA synthesis and adapter ligation, the library was amplified with 12 rounds of PCR cycles. The enriched and cleaned libraries were pooled and loaded on the sequencer. The obtained FASTQ files and quantitative results are available from the GEO DataSets database (GSE174586).

### RNA Sequencing Data Analysis

RNA sequencing was performed to study changes in gene expression in the corneal endothelium of *Slc4a11* KO mice versus WT at 12 weeks of age. The study identified 806 genes with gene expression increased in KO versus WT more than 50% (FC > 1.5) and 644 genes with gene expression decreased in KO versus WT more than 50%.

### QPCR

Total RNA was isolated from a pool of six corneal endothelium-Descemet's membranes (CEDM) peeled with microforceps from three mice per genotype using RNeasy Micro kit (Qiagen #74004). DNA was removed from the sample using AllPrep AP DNA columns (Qiagen #80204). Three pools of RNA were obtained per genotype. Two hundred nanograms of RNA were converted to cDNA using High Capacity RNA-to-cDNA Kit (Applied Biosystems #4387406). QPCR was performed in a 25 µl mixture containing 1 ng of cDNA, 12.5 µl of Brilliant II SYBR Green QPCR Master Mix (Agilent # 600828), 0.375 µl of ROX reference dye (diluted 1:500) and 200 nM of each primer. Each of the six samples were run in triplicate. The cycling parameters were as follows: initial denaturation for 10 minutes at 95°C, followed by 40 cycles of 30 seconds denaturation at 95°C, one minute annealing at 55°C, and 30 seconds extension at 72°C. This was followed by a melting curve to analyze the presence of a single product: one minute at 95°C, 30 seconds at 55°C, and 30 seconds at 95°C. The threshold cycles (Ct) were calculated with MxPro software (Agilent). The expression was normalized against the housekeeping gene β-actin. Fold change of KO versus WT was obtained using the 2-∆∆CT method. The results are presented as log_2_ fold change.

### Western Immunoassay

The two corneal endothelial peelings of one animal were pooled and lysed in 25 µl RIPA lysis buffer containing 1 mM PMSF. Five microliters were used to measure protein by BCA method. Equal amounts of protein (1.5 µg) were loaded into the wells of the 12 to 230 kDa separation module of a Protein Simple Wes system (Protein Simple, San Jose, CA, USA) and analyzed following manufacturer's instructions. Antibodies were added in the following dilutions: MCT1 (Santa Cruz Biotechnologies #sc-365501) 1:50, MCT2 (Santa Cruz Biotechnologies #sc-166925) 1:10, MCT4 (Ab Clonal #A10548) 1:10, and α-Tubulin (Novus Biologicals #NB100-690) 1:10. Secondary antibodies and substrate were provided by the Wes kit. Wes data are obtained as virtual blots in which the molecular weight and signal intensity are presented. Results in the form of traditional electropherograms are also obtained with this approach.

### Lactate Levels

Corneas were dissected at the limbus and epithelium and endothelium were removed. Stromas were placed in preweighed Eppendorf tubes and pulverized in liquid nitrogen and homogenized in 30 µl of PBS using plastic disposable pestle. The sample was centrifuged at 15,000 *g* for 15 minutes at 4°C. The supernatant was recovered. The remaining pellet was dried at 60°C within a vacuum centrifuge for two hours and weighed (dry weight). Lactate was measured in the supernatant using Fluorescent kit (Biovision #K607) according to manufacturer's instructions.

### ATP Levels

Corneas were obtained and CEDM was peeled with fine forceps. Two CEDM from the same mouse were combined and pulverized in liquid nitrogen and homogenized in 30 µl of RIPA buffer plus 1 mM PMSF using plastic disposable pestle. The sample was centrifuged at 15,000 *g* for 15 minutes at 4°C. The supernatant was recovered. ATP was measured by bioluminescence assay (Thermo Fisher Scientific #A22066) according to manufacturer's instructions. Protein was measured by BCA method.

### Na/K ATPase Activity

Six CEDM (6 mice) were pooled and homogenized in 130 µl of assay buffer provided by Na^+^/K^+^-ATPase activity kit (#K417-100, BioVision) using plastic disposable pestle. The sample was sonicated and then centrifuged at 10,000 *g* for 10 minutes. The supernatant was recovered and phosphates in the sample were depleted by incubating with 40 µl of PiBind resin (#501-0015, Innova Biosciences) for 15 minutes at room temperature in a rotary device. After centrifugation at 1,000 *g* for two minutes the sample was recovered, and ATPase activity was measured in 5 µl of sample in the presence or absence of 1 mM ouabain. Na^+^/K^+^-ATPase activity was obtained by subtracting the activity in presence of ouabain from the total activity. Protein was measured by BCA method.

### Endothelium Permeability Assay

Whole corneas were dissected and placed in small dimpled holders endothelial side up. A volume of 10 µl of 0.1% sodium fluorescein in bicarbonate-rich Ringer (BR) was added on the endothelial side and incubated at room temperature for 30 minutes. The corneas were washed three times for five minutes each with 10 µl BR. After removing all liquid, corneas were positioned in wells of a 96-well plate and fluorescence (excitation: 485 nm; emission: 520 nM) was measured in a microplate reader. As a positive control, corneas were incubated for one hour with 10 µl of EGTA 2 mM in Ca-free PBS prior to incubation in fluorescein.

### Statistical Analysis

Three or more replicates of each experiment was performed. Error bars represent ± SEM. Statistical analysis was performed using GraphPad Prism 9.0 (GraphPad Software, Inc.). Student's *t*-test was used for two-group comparison. One-way ANOVA with post hoc Tukey multiple comparison test was used for data with more than two groups. Significance was defined as *P* < 0.05.

### Raw Data

Full western blots and RNA-seq analysis are provided in Supplementary Files S1 and S2.

## Results

There are no unique corneal endothelial promoters, so we used a simple whole-body inducible KO, as in the conventional *Slc4a11* KO.^6^^,^^9^ Inducible *Slc4a11* knockout was performed by feeding Tm to 8-week old *Slc4a11^Flox/^^Flox^*//Rosa*^CreERT2^*^/^*^CreERT2^* mice (Fig. 1A and Methods). Tm induction resulted in a significant decrease of *Slc4a11* transcript levels at four weeks posttreatment. (Relative quantity, control: 1.00 ± 0.11 versus Tm: 0.02 ± 0.005, *P* = 3.07 × 10^−8^, *n* = 4) (Fig. 1B). With Tm treatment, significant increase in corneal thickness was observed at two weeks. The corneal thickness continued to increase with nearly 50% increase evident at eight weeks (Figs. 1C and D). There was no significant change in cell density (control: 2648 ± 137 cells/mm^2^ versus Tm: 2520 ± 211, *P* = 0.39, *n* = 3), cell area (control: 379 ± 20 µm^2^ versus Tm: 401 ± 37, p = 0.38, n = 3), or % polymegathism (control: 16.2 ± 2.4 % versus Tm: 16.5 ± 4.0, *P* = 0.92, *n* = 3) at eight weeks postinduction; however, cell shape was altered as percentage hexagonality was significantly decreased (control: 62.9 ± 2.7% versus Tm: 52.3 ± 3.7, *P* = 0.005, *n* = 3) (Figs. 1E and F). Corneal neovascularization was absent as in the conventional KO. As a control of potential nonspecific effects of tamoxifen on corneal thickness, WT mice at 10 weeks of age were fed Tm for 14 days. No change in corneal thickness was found (CT initial: 111.53 ± 3.35 µM versus CT final: 111.89 ± 3.25, *P* = 0.285, *n* = 6).

**Figure 2.**
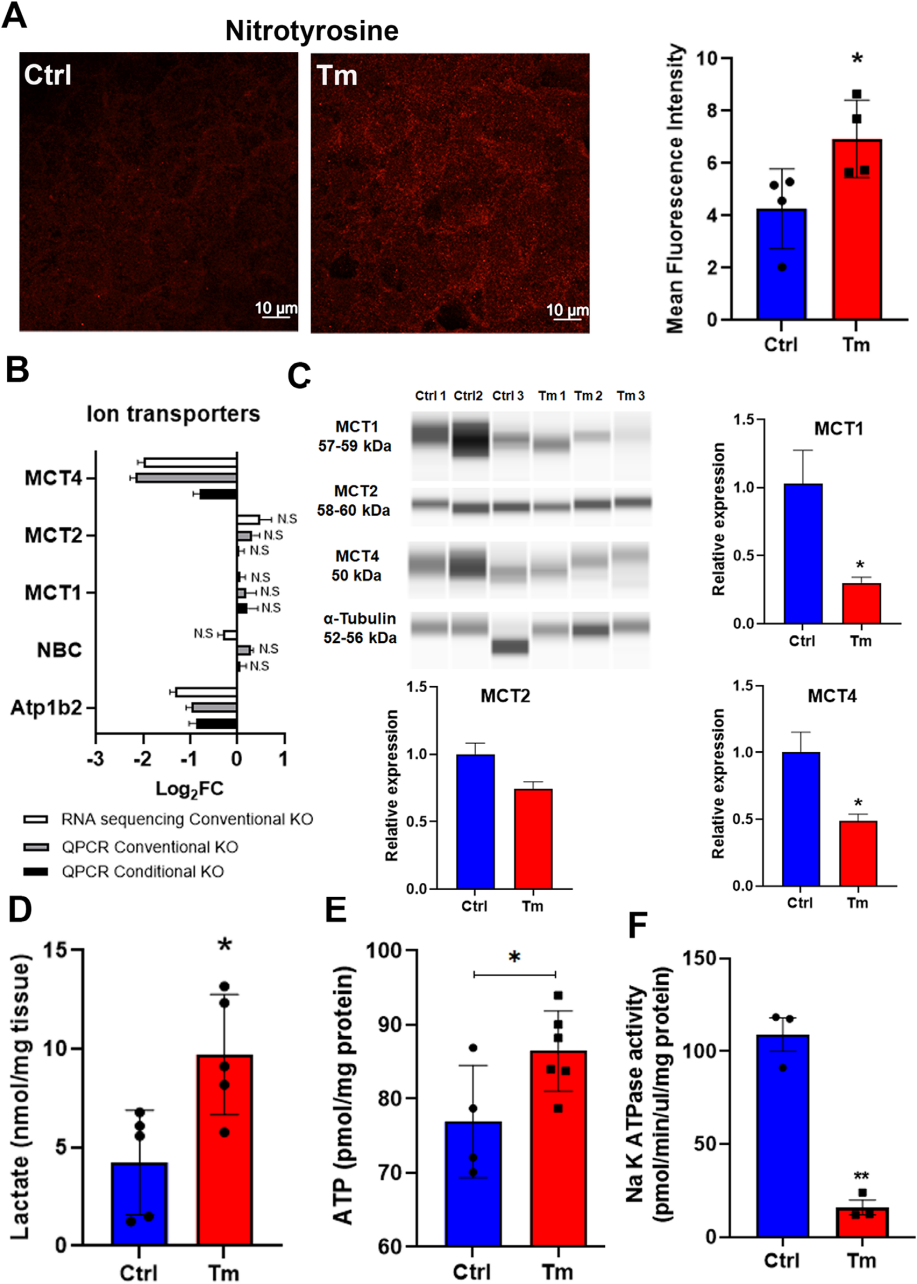
Inducible *Slc4a11* KO triggers oxidative stress damage, gene expression changes, and disruption of the endothelial pump function. **A**. Left panel: nitrotyrosine staining of corneal endothelium from control and Tm treated mice at eight weeks. Right panel: mean fluorescence intensity; mean ± SEM, *n* = 4, *P < 0.05. **B**. Ion transporters expression in conditional KO versus Ctrl (*n* = 3), and conventional KO by QPCR (*n* = 3), and RNA sequencing (*n* = 5). Conventional KO corresponds to mice of three months of age, *P < 0.05. **C**. Wes immunoassay of MCT1, MCT2, and MCT4 and α-Tubulin; mean ± SEM (*n* = 3), *P < 0.05. **D**. Stromal lactate content; mean ± SEM (*n* = 5), *P < 0.05. **E**. ATP content; mean ± SEM, *n* = 4 for Ctrl, *n* = 6 for Tm, *P < 0.05. **F**. Na K ATPase activity, mean ± SEM, *n* = 3, *P* < 0.01. All conditional KO data is after eight weeks of Tm treatment.

A hallmark of corneal endothelial dystrophies and the *Slc4a11* KO CHED model is increased oxidative stress within corneal endothelium.^9^^,^^14^^,^^15^ We therefore examined nitrotyrosine levels in the endothelium as a measure of oxidative damage of proteins. Tm treated mice showed significantly increased nitrotyrosine staining at eight weeks of treatment relative to control (Fig. 2A).

The formation of corneal edema in the early days of the inducible KO concomitant with normal cell density implicates changes in expression of transport related proteins or availability of ATP needed for transport function. Preliminary RNA-seq analysis of conventional KO endothelium suggested decreased lactate transporter and Na^+^/K^+^-ATPase subunit Atp1b2 expression. Figure 2B QPCR shows that the ion transporters MCT4 and Atp1b2 subunit of the Na^+^/K^+^-ATPase were significantly downregulated in the conditional KO similar to the conventional. Interestingly, protein levels of MCT1 and MCT4 were significantly decreased while MCT2 was not changed (Fig. 2C).

Corneal endothelial pump function is linked to corneal lactate efflux that is dependent on Na^+^/K^+^-ATPase activity.^8^ Therefore, we examined stromal lactate, ATP availability, and Na^+^/K^+^-ATPase activity. Corneal lactate levels of Tm treated mice were more than twofold higher than control mice at eight weeks post Tm (Fig. 2D). ATP levels in Tm treated mice were 12% higher than control (Fig. 2E). Whereas Na^+^/K^+^-ATPase activity of the corneal endothelium was decreased sevenfold in Tm versus Ctrl (Fig. 2F). Therefore, the resultant corneal edema, consistent with the accumulation of lactate, was not due to availability of ATP. The diminished expression of lactate transporters (Figs. 2B–C) and the very low Na^+^/K^+^-ATPase activity combined can explain the accumulation of lactate.

**Figure 3.**
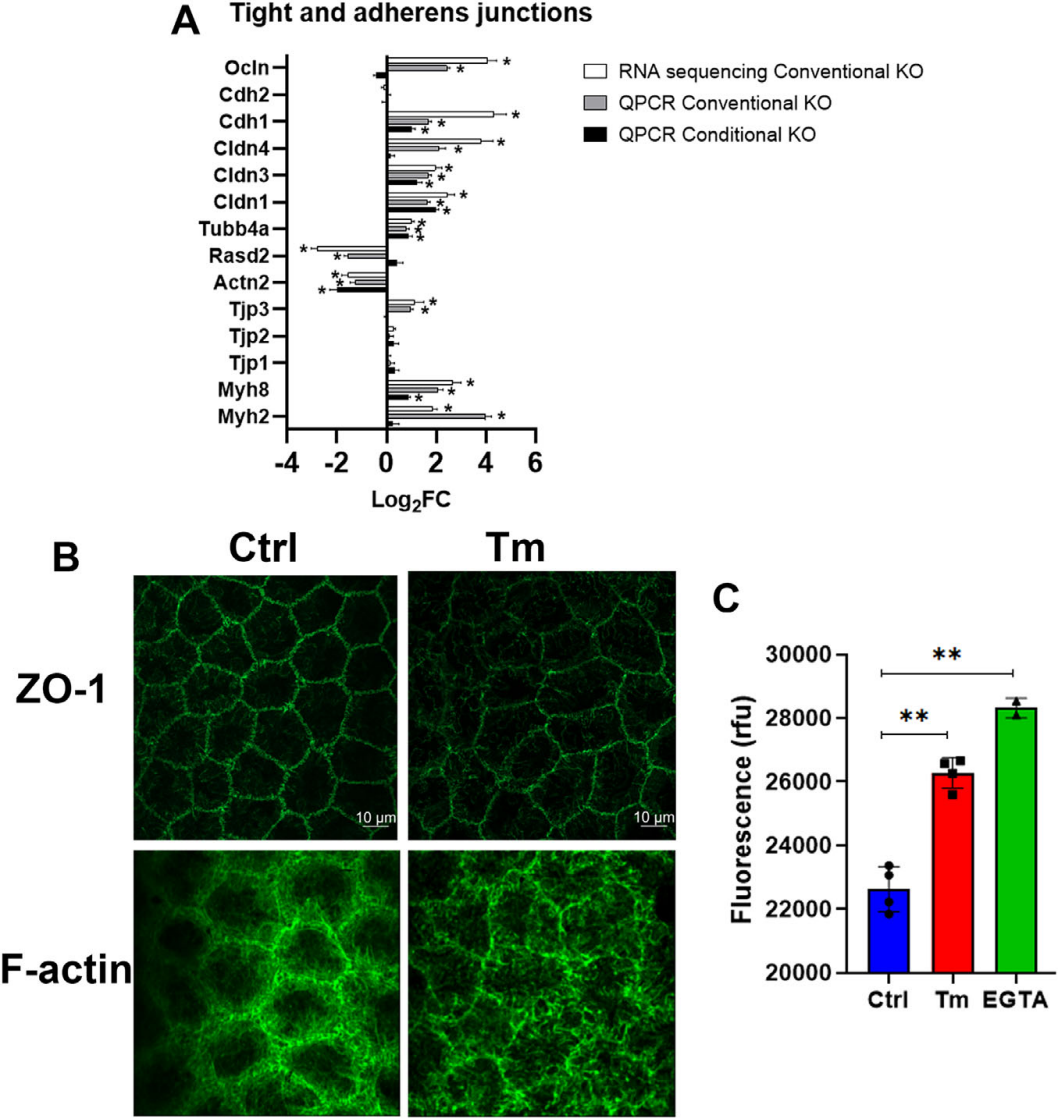
Inducible *Slc4a11* KO disrupts the endothelial barrier function. **A.** Tight and adherens junction gene expression (same conditions as Figure 2B). **B**. Representative image of ZO-1 and F-actin. **C**. Endothelial fluorescein permeability; mean ± SEM, *n* = 4 for Ctrl and Tm, *n* = 2 for EGTA, **P < 0.01. All conditional KO data is after eight weeks of Tm treatment.

The endothelial pump also requires an intact osmotic membrane to link lactate flux with water flux.^7^ In this regard, cell morphology is altered in the conventional *Slc4a11* KO,^6^ and we found that hexagonality was altered after eight weeks of conditional KO (Fig. 1). Therefore, we more closely examined cell junctions. The tight and adherens junctions proteins Cldn1, Cldn3, Myh8, and Tubb4a were upregulated, whereas Actn2 was downregulated; suggesting alterations in junctions and cortical cytoskeleton (Fig. 3A). Actn2 acts as a bridge between Cdh1 and Cdh2 (E-cadherin and N-cadherin) and the actin cytoskeleton. Tight junction structure was compromised in *Slc4a11* KO as observed by ZO1 staining (Fig. 3B, upper panel). Cortical cytoskeleton (F-actin) associated with adherens junctions was clearly disrupted (Fig. 3B, lower panel). Consistent with alterations in junction structure and junctional gene expression, paracellular permeability of corneal endothelial cells increased by 16% in Tm versus Ctrl mice (Fig. 3C), indicating that *Slc4a11* KO leads to junctional alterations and compromised barrier function.

**Figure 4.**
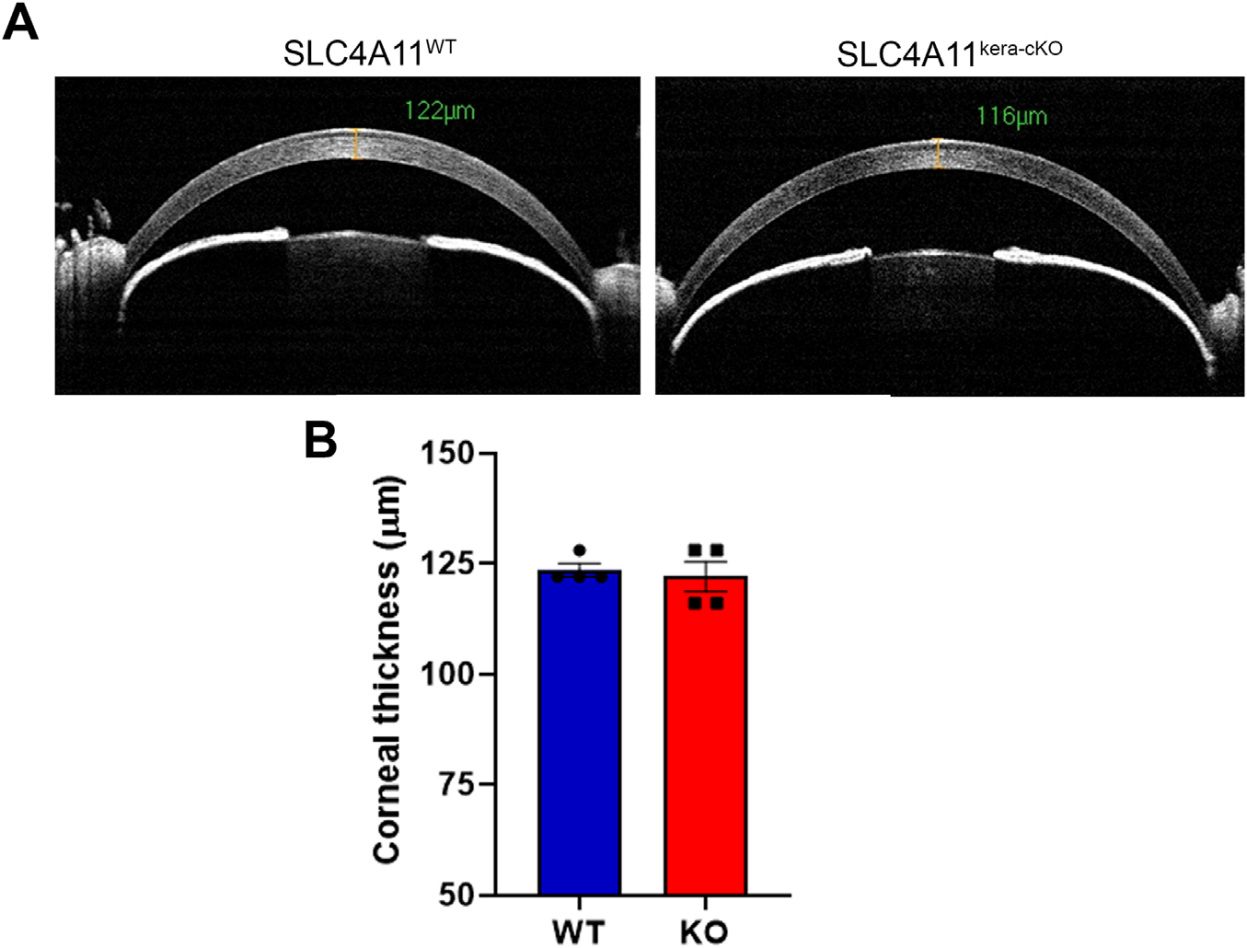
Keratocyte *Slc4a11* activity does not contribute to corneal thickness. **A.** Representative WT and keratocyte-specific *Slc4a11* KO OCT images at three months of age. **B**. Corneal thickness, mean ± SEM, *n* = 4, *P* = 0.71.

Slc4a11 is highly expressed in corneal endothelium. A previous report indicated that Slc4a11 is also expressed in corneal keratocytes.^16^ As our conditional *Slc4a11* KO is a whole-body KO and endothelial specific promoters are not known, we took advantage of keratocyte specific knockout and asked if there was any effect on corneal thickness to rule out potential keratocyte effects. In keratocyte only *Slc4a11* conditional KO, no significant differences were found in corneal thickness (Kera-cKO: 122 ± 3.46 µm versus WT: 123.5 ± 1.50, *P* = 0.71, *n* = 4) indicating that corneal keratocyte Slc4a11 activity does not play a role in maintaining corneal thickness (Figs. 4A and B).

## Discussion

The conventional *Slc4a11* KO shows progressive corneal edema that is apparent at eye opening, altered endothelial morphology, significant endothelial oxidative damage, and mitochondrial dysfunction, with eventual cell loss and diminished cell density.^6^^,^^9^ This recapitulates the phenotype of CHED and has provided many valuable insights to understanding this corneal endothelial dystrophy. However, because the loss of *Slc4a11* is present throughout development, the initial cellular events that lead to the phenotype and ultimate understanding of the direct and indirect functions of Slc4a11 are obscured. Therefore, an inducible KO can provide a window of opportunity to examine these events. This initial report describes creation of a *Slc4a11* inducible KO that shows development of a similar phenotype as the conventional KO, thereby validating the model and providing a tool for further use in examining events with finer time intervals or use of noninvasive in vivo physiological probes.

The advantages of the inducible model will be best utilized by incorporating noninvasive measures of cell function. Here we used OCT to measure development of corneal edema, which could be done at smaller intervals. Advances in noninvasive imaging of the mouse cornea could also be used to examine changes in endothelial morphology over time. Similarly, there are several genetically encoded fluorescent probes available to measure different kinds of reactive oxygen species.^17^ These probes can be virally transfected into endothelial cells before KO induction. In this way, development of specific kinds of ROS can be visualized over time within endothelial cells. In addition, crossing the inducible KO with transgenic models harboring fluorescent physiological indicators can be performed making this model a powerful tool for uncovering the earliest cellular events in vivo.

The phenotype of edema concomitant with changes in endothelial morphology without a decrease in cell density seen in the conditional KO is similar to the one observed in early stages (10–12weeks old) of the conventional KO model.^6^^,^^7^ In both models we found evidence of oxidative stress-mediated damage^9^ and alteration of the corneal endothelial pump function as measured by increased lactate accumulation in the stroma concomitant with corneal edema.^7^ The increased corneal lactate could be due to the lack of MCT1 and 4 and/or reduction in Na^+^/K^+^-ATPase expression and activity. MCT1 protein expression in corneal endothelium appears to be regulated posttranscriptionally as no changes are observed at the mRNA level. Knockdown of MCT1 and MCT4 in the in vivo rabbit leads to corneal edema and lactate accumulation;^18^ indicating that MCT downregulation by itself is sufficient to cause edema. Similarly, pharmacological inhibition of MCTs causes corneal swelling and lactate accumulation in the in vitro rabbit cornea.^19^ However, all secondary active transport processes, including MCT transport, are dependent on primary active transport, that is, the Na^+^/K^+^-ATPase, that controls the ion gradients and pH regulation that drives secondary transport. In this model we found downregulation of the Na^+^/K^+^-ATPase subunit Atp1b2 and a sevenfold decrease in enzyme activity. Therefore, it is likely that the lactate accumulation is due to a combination of downregulation of MCTs and Na^+^/K^+^-ATPase.

Slc4a11 is found in the basolateral membrane of corneal endothelium as well as multiple cytoplasmic locations,^20^^,^^21^ including the mitochondrial inner membrane where it functions as an ammonia sensitive mitochondrial uncoupler that facilitates the catabolism of glutamine and anaplerosis.^9^ In the presence of glutamine, lack of Slc4a11 leads to increased production of superoxide that damages mitochondria. We have reported^9^ and more recently Zhang et al.^11^ have observed that the altered metabolism of a corneal endothelial cell line derived from the *Slc4a11* KO mouse leads to significantly diminished ATP production. If corneal edema and lactate accumulation were a result of energy deficiency, one would expect a significantly lower ATP content in KO. Paradoxically, we found increased ATP content in the corneal endothelial tissue from the inducible KO versus WT. Since the Na^+^/K^+^-ATPase is the major consumer of ATP in the cell,^22^ these results suggest that the corneal endothelium of the *Slc4a11* KO both produces and consumes less ATP than WT. The possibility of a nonspecific effect of tamoxifen is unlikely. Tamoxifen is fed for only two weeks, and the endpoint KO effect is seen at eight weeks when any potential side effects would have long dissipated. Moreover, WT mice fed with tamoxifen showed no change in corneal thickness indicating that there was no effect on basic metabolic activity. Importantly, the observed increase in stromal lactate, CE ATP, as well as decreased Na^+^/K^+^-ATPase activity is observed in the conventional KO (unpublished results, manuscript in preparation).

In addition to the presence of energized membrane transporters, the lactate-linked endothelial pump requires an intact membrane barrier to achieve osmotic water flux.^7^^,^^23^ We found that Actn2 is downregulated in *Slc4a11* KO corneal endothelium. Actn2 acts as a bridge between Cdh1 and Cdh2 (E-cadherin and N-cadherin) and actin cytoskeleton. This may contribute to the perturbation of cell junctions. In KO we observed increased permeability to fluorescein along with upregulation of several junctional genes especially claudins, which may represent a futile repair response as seen in intestinal epithelium after loss of barrier function due to inflammation.^24^

Lastly, we found that even though Slc4a11 is expressed in keratocytes,^16^ this activity is not secondarily involved in the formation of corneal edema, for example, by altering glycosaminoglycan production. This is consistent with the central role of endothelial Slc4a11 in pump maintenance by reducing oxidative stress^9^^,^^14^ and facilitating glutamine catabolism. Oxidative stress is likely the trigger for altered transporter gene expression in this model. Inhibiting superoxide production by circumventing glutaminolysis significantly reduces corneal edema in the conventional KO in vivo^9^. The mechanisms by which mitochondrial dysfunction and superoxide excess lead to the alterations in transporter expression and perturbation of the barrier function seen here remain to be determined.

## Supplementary Material

Supplement 1

Supplement 2
