# Supplementary figures and images for: Inducible Slc4a11 Knockout Triggers Corneal Edema Through Perturbation of Corneal Endothelial Pump
Source: Invest Ophthalmol Vis Sci. 2021 Jun 30;62(7):28. doi: 10.1167/iovs.62.7.28 (PMC8826551; doi:10.1167/iovs.62.7.28)

# $\alpha$ -Tubulin. Band 52-56 kDa was analysed

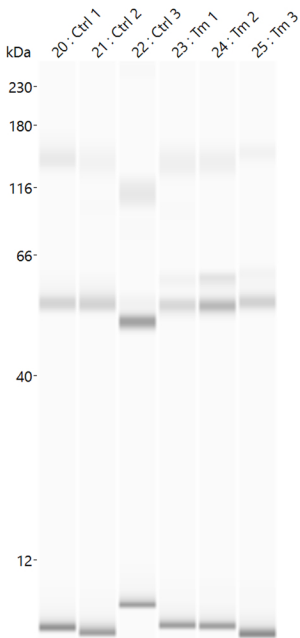

# MCT1

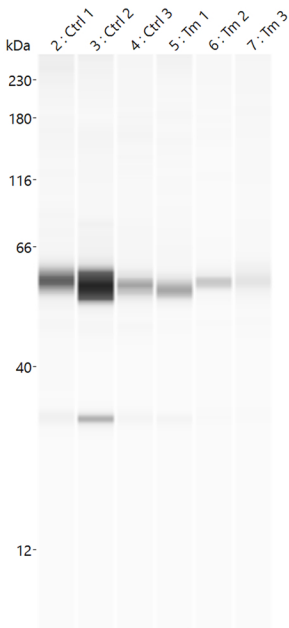

## MCT2. Lower MW band was analyzed

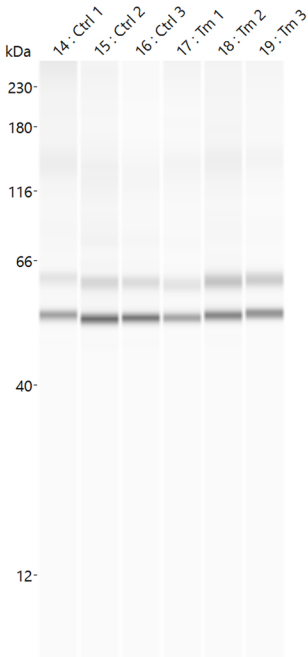

# MCT4

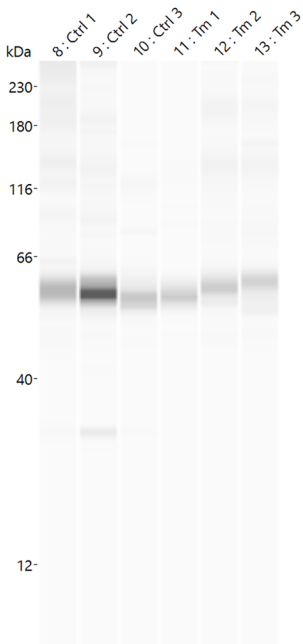

Supplement: Supplement 1 [file iovs-62-7-28_s001.pdf]
